# Tocotrienol Attenuates Stress-Induced Gastric Lesions via Activation of Prostaglandin and Upregulation of COX-1 mRNA

**DOI:** 10.1155/2013/804796

**Published:** 2013-07-22

**Authors:** Mohd Fahami Nur Azlina, Yusof Kamisah, Kien Hui Chua, Hj Mohd Saad Qodriyah

**Affiliations:** ^1^Department of Pharmacology, Faculty of Medicine, Universiti Kebangsaan Malaysia, Jalan Raja Muda Abdul Aziz, 50300 Kuala Lumpur, Malaysia; ^2^Department of Physiology, Faculty of Medicine, Universiti Kebangsaan Malaysia, Jalan Raja Muda Abdul Aziz, 50300 Kuala Lumpur, Malaysia

## Abstract

The present study aims to distinguish the effect of tocotrienol on an important gastric protective factor, prostaglandin E_2_ (PGE_2_), in stress-induced gastric injury. Twenty-eight Wistar rats were divided into four groups of seven rats each. Two control groups were fed commercial rat diet, and two treatment groups were fed the same diet but with additional dose of omeprazole (20 mg/kg) or tocotrienol (60 mg/kg). After 28 days, rats from one control group and both treated groups were subjected to water-immersion restraint stress for 3.5 hours once. The rats were then sacrificed, their stomach isolated and gastric juice collected, lesions examined, and gastric PGE_2_ content and cyclooxygenase (COX) mRNA expression were determined. Both the regimes significantly attenuated the total lesion area in the stomach compared to the control. Gastric acidity, which was increased in stress, was significantly reduced in rats supplemented with omeprazole and tocotrienol. The PGE_2_ content was also significantly higher in the rats given tocotrienol supplementation compared to the control followed by an increase in COX-1 mRNA expression. We conclude that tocotrienol supplementation protected rat gastric mucosa against stress-induced lesions possibly by reducing gastric acidity and preserving gastric PGE_2_ by increasing COX-1 mRNA.

## 1. Introduction

The reported incidence of stress-related mucosal damage varies from 6% to 100% in critically ill patients [1]. The pathological basis for the development of stress-induced gastric lesions has been postulated to be multifactorial. These include changes in the gastric acid secretion [2], reduction of gastric mucosal blood flow [3], inhibition of gastric mucus and bicarbonate secretion [4], and inhibition of gastric mucosal prostaglandin synthesis [5].

Prostaglandins are generated in the gastric mucosa via the activity of the enzyme cyclooxygenase (COX). It exists in two genetically different isoforms, constitutive COX-1 forms and inducible COX-2 [6]. COX-1 had been shown to exhibit cytoprotective effects on gastric mucosa whereas COX-2 had been implicated in the inflammatory reactions and tissue damage involving various cytokines, endotoxins, and growth factors [7, 8]. 

In the gastric microenvironment, prostaglandins (PG) are believed to maintain the integrity of the gastric mucosa by stimulating secretions of mucus and bicarbonate and modulating mucosa blood flow [9, 10]. Prostaglandin E_2_ (PGE_2_) effects on gastrointestinal tract include inhibition of gastric acid secretion, contraction of longitudinal muscle, and relaxation of circular muscle [11]. Previous studies found that PGE_2_ level after 3.5 hours of water-immersion restraint stress was decreased by at least half of its normal concentration, with a significant formation of lesions [12]. In a preclinical research setting, the use of animal models to mimic the damage to the gastric mucosa due to stress in the critically ill is well established [5]. Immobilization or restraint has been used repetitively because of its ability to produce and reproduce stress ulcers.

Oxidative stress had been implicated in the formation of stress-induced gastric lesions [2, 13, 14]. Supplementation with vitamin E, both tocopherol and tocotrienol [2, 9, 14], has been demonstrated to prevent gastric mucosal development in rats exposed to stress. Both possess antioxidant properties, but tocotrienol was reported to possess better antioxidant capability than tocopherol [15]. Hence, a compound with pure antioxidant property may be potentially useful in minimizing gastric mucosal damage caused by free radicals. Among various stressors used in animals, water-immersion restraint stress [6, 7] demonstrated the most reproducible results, which led to the formation of gastric lesions.

Tocotrienol is a minor plant constituent, abundant in palm oil and cereal grains, which can provide a significant source of vitamin E-like activity. In this study, we used vitamin E that was extracted from palm oil, which contains approximately 90% tocotrienol. The present study aimed to investigate the effects of tocotrienol on gastric cyclooxygenase mRNA expression level, gastric acidity, gastric PGE_2_, and gastric lesions in rats exposed to a water-immersion restraint stress. This study also compared the effects of tocotrienol with omeprazole, a commonly used antiulcer drug on the parameters measured.

## 2. Material and Methods

Male *Wistar* rats (*n* = 28) were divided into four equally sized groups. Two control groups were fed a normal rat diet (NS and S) while the treatment groups received the same diet but with oral supplementation of tocotrienol (TT) or omeprazole (OMZ) at 60 mg/kg and 20 mg/kg body weight, respectively, for 28 days. The dose chosen was based on our previous studies which had shown a protective effect of tocotrienol on stress-induced gastric lesions [2, 16]. Tocotrienol and omeprazole were given in vitamin-free palm oil, which acted as a vehicle and was administered by an oral gavage using an 18 G gavage needle. Both control groups were sham-administered with vitamin-free palm oil. At the end of the treatment period, the rats from one control group (stressed control) and both of the treated groups were exposed to water-immersion restraint stress. After the exposure to stress, the rats were sacrificed. The dissected stomachs were taken for evaluation of gastric lesion, gastric acidity, prostaglandin E_2_ level, and cyclooxygenase mRNA expression level. 

All rats were kept on a regular night/day cycle, with natural light for a period of 10 hours (0700 to 1700 h). Throughout the feeding period, all rats were habituated to handling the reduction of their stress-related disturbances. The rats were housed in large cages with wide wire-mesh bottoms to prevent coprophagy. Food and water were given ad libitum throughout the experiment. Prior ethical approval was obtained from the University Kebangsaan Malaysia Animal Ethics Committee (UKMAEC). 

Vitamin E used in this study contained 44.8% d-*γ*-tocotrienol, 29.4% d-*α*-tocotrienol, 10.8% d-*δ*-tocotrienol, and 5% d-*β*-tocotrienol, extracted from palm oil, which is referred to as tocotrienol in this paper. The tocotrienol was obtained from Carotech Sdn Bhd (Malaysia). Tocotrienol had been shown to be well tolerated where Oo et al. [17] found that palm oil extract (containing 80% tocotrienols) administered to young rats and mice at a dose up to 25000 mg/kg body weight in a subchronic toxicity study (30 days daily dosing) showed no appreciable adverse effect in animals with respect to physical manifestations or behavioral changes.

Rats were restrained by placing them in individual plastic restrainers, measuring approximately 17 × 5 cm, and immersing them in water neck deep for 3.5 hour once, as previously described by Kamisah et al. [13]. Following the restraining procedure, the rats were sacrificed; the stomachs were dissected along the greater curvature and examined for lesions.

### 2.1. Macroscopic Assessment of Stress-Induced Gastric Lesions

The macroscopic assessment of stress-induced gastric lesions in the gastric mucosa was performed by two independent examiners who were blinded to the treatment that the rats received. The assessment of lesions was done according to a semiquantitative scale. The scale used was as follows: 5 = presence of gastric erosion and generalized hemorrhage covering more than 80% of the gastric mucosa, 4 = presence of gastric erosion and hemorrhage covering 50%–80% of the gastric mucosa, 3 = presence of gastric erosion and hemorrhage covering 30%–50% of the gastric mucosa, 2 = hemorrhage covering 10%–30% of the gastric mucosa, 1 = generalized erythema with present of hemorrhage, and 0 = no visible lesion. 

### 2.2. Determination of Gastric Acidity

Measurement of the gastric acidity was done following a method described by Shay et al. [18]. The junctions between the stomach/esophagus and duodenum/pylorus were secured before the stomach was isolated. Then 3 mL of distilled water was introduced into the stomach, and the organ was carefully shaken. The gastric juice was then collected and centrifuged for 10 minutes at 3000 rpm. The supernatant was taken and diluted 10 times. Following this, a few drops of phenolphthalein were added to the solution. Titration was done using 0.01 M solutions until the color of the test solution changed to light pink indicating pH 7.0. The volume of sodium hydroxide (NaOH) needed in the titration was used for the calculation to derive the hydrogen ion concentration. 

### 2.3. Measurement of Gastric Prostaglandin E_2_ Content

Sample preparation for prostaglandin E_2_ (PGE_2_) assay was done using the method previously described by Redfern et al. [19]. Gastric PGE_2_ content was measured using EIA kit (514010, Cayman, USA).

### 2.4. Cyclooxygenase mRNA Quantitation

For the COX-1, COX-2, and GAPDH mRNA quantitation, the standard QuantiGene Plex 2.0 assay kit (Genospectra, Fremont, CA, USA) protocol was followed. Briefly, the tissue lysate was transferred to a capture well in the presence of the gene-specific probe set and then hybridized at 53°C overnight. Wells were washed twice with bDNA wash buffer and then incubated at 46°C sequentially with an amplifier and an alkaline phosphatase-linked label probe with a wash step between the incubations. After the final wash step, the addition of streptavidin phycoerythrin (SAPE) generated a signal that was proportional to the amount of target RNA present in the sample. The luminescence signal was detected using a Luminex instrument. The protocol followed was as previously described by Zhang et al. [20].

### 2.5. Statistical Analysis

Statistical analysis was carried out using the PRISM software version 6.00 (Graphpad, San Diego, CA, USA). All data were normally distributed. The results are expressed as mean ± SEM. Statistical significance (*P* < 0.05) was determined by ANOVA and Tukey's post hoc test (parametric).

## 3. Results

**Figure 1:**
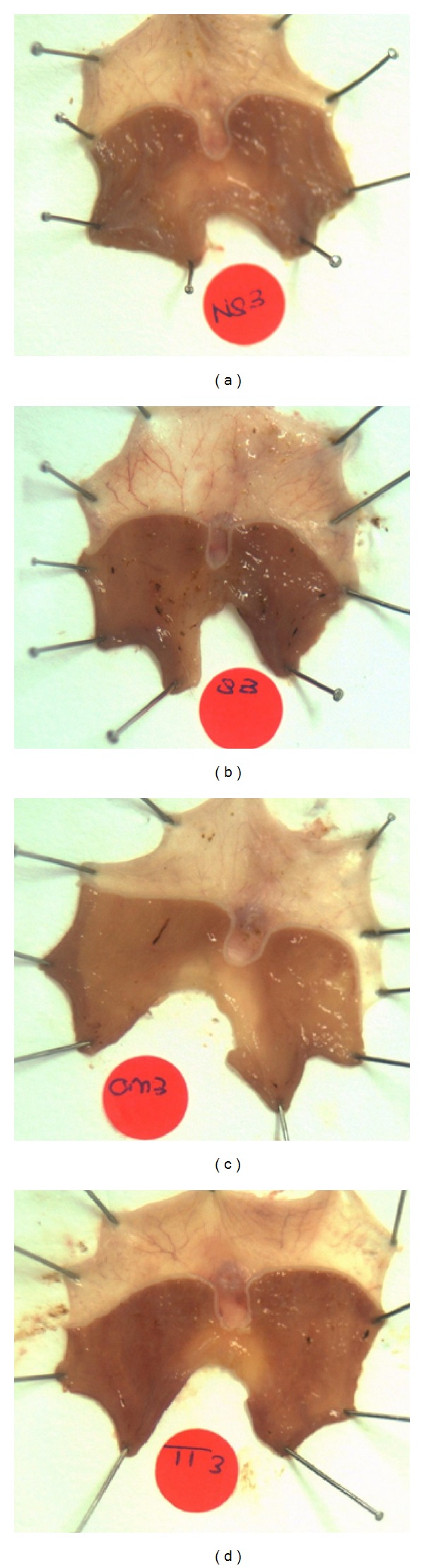
Macroscopic observation of water-immersion restraint stress- (WIRS-) induced gastric lesions. (a) Gastric tissue of normal rat (no lesions). (b) Gastric tissue of a rat exposed to 3.5 h of WIRS (developed gastric ulcer). (c) Gastric tissue of a rat exposed to 3.5 h of WIRS with omeprazole (OMZ) supplementation. (d) Gastric tissue of a rat exposed to 3.5 h of WIRS with tocotrienol (TT) supplementation.

**Figure 2:**
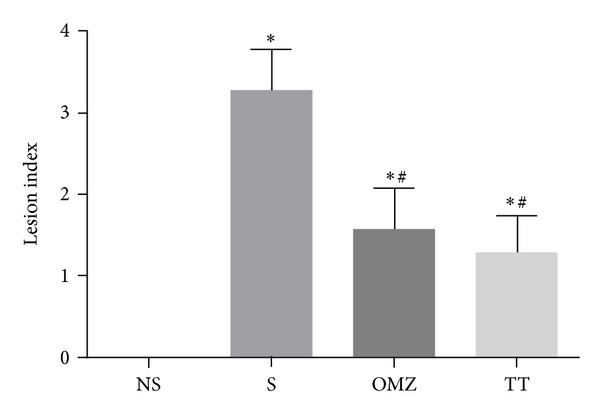
Effects of tocotrienol and omeprazole on gastric lesions in rats exposed to water immersion restraint stress (*n* = 7). *versus nonstressed control (NS) (*P* < 0.05). ^#^versus stressed control (S) (*P* < 0.05).

Rats exposed to water-immersion restraint stress for 3.5 hours showed presence of considerable ulcers in the form of gastric erosion and hemorrhagic mucosal lesions confined to the corpus (glandular part of the stomach) (Figure 1). As shown in Figure 2, the gastric lesion index in the stressed control (S) group was higher by 60.9% compared to the TT group (*P* = 0.0081) and 52.2% compared to OMZ group (*P* = 0.0265). These findings indicate that both tocotrienol and omeprazole were able to reduce the formation of stress-induced gastric lesions. Rats killed after 28 days of feeding period and not exposed to stress had no gastric mucosal lesion. The gastric lesion index in the control group was 8.4-fold higher (*P* < 0.0001) compared to the nonstressed rats in the same group (*F*(3,24) = 11.56, *P* < 0.0001).

**Figure 3:**
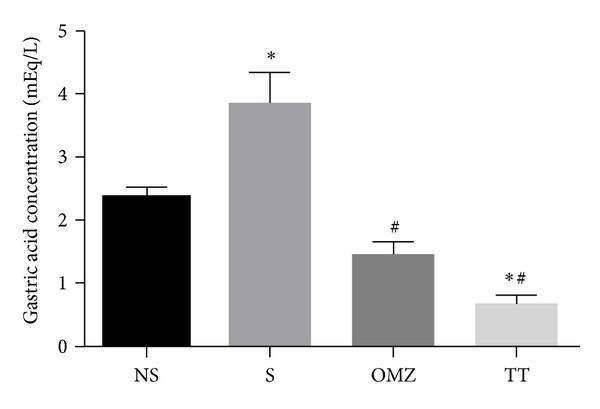
Effects of tocotrienol and omeprazole on gastric acidity in rats exposed to water immersion restraint stress (*n* = 7). *versus nonstressed control (NS) (*P* < 0.05). ^#^versus stressed control (S) (*P* < 0.05).

As shown in Figure 3, the gastric acidity in the control stressed group was increased by 42% (*P* = 0.003) compared to the nonstressed control. Gastric acidity of TT (*P* < 0.0001) and OMZ (*P* < 0.0001) stressed groups was significantly reduced compared to the stressed control. We found no significant difference between the gastric acidity levels in the TT and the OMZ stressed groups (*F*(3,24) = 29.24, *P* < 0.0001). 

**Figure 4:**
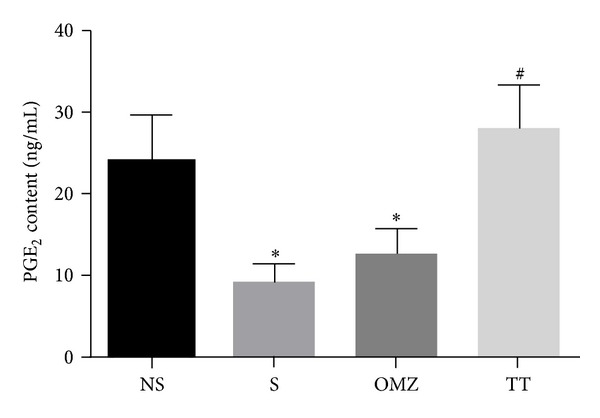
Effects of tocotrienol and omeprazole on gastric prostaglandin E_2_ content in rats exposed to water immersion restraint stress (*n* = 7). *versus nonstressed control (NS) (*P* < 0.05). ^#^versus stressed control (S) (*P* < 0.05).

The mean gastric PGE_2_ content in rats exposed to restraint stress was significantly lower (*P* = 0.0097) compared to the nonstressed control, as shown in Figure 4. The findings suggest that stress altered the gastric PGE_2_ content. Supplementation of TT increased PGE_2_ content compared to both nonstressed (*P* = 0.05) and stressed groups (*P* = 0.01) (*F*(3,24) = 5.954, *P* = 0.0021). Prostaglandin E_2_ content was maintained towards the normal level similar to the nonstressed rats. This was not observed in the omeprazole-treated rats; no significant change was observed between the omeprazole and the nonstressed control (*P* > 0.05). The finding suggests that the protective effect of TT could partly be due its ability to increase the gastric PGE_2_ content in stress and is irrespective of stress.

**Figure 5:**
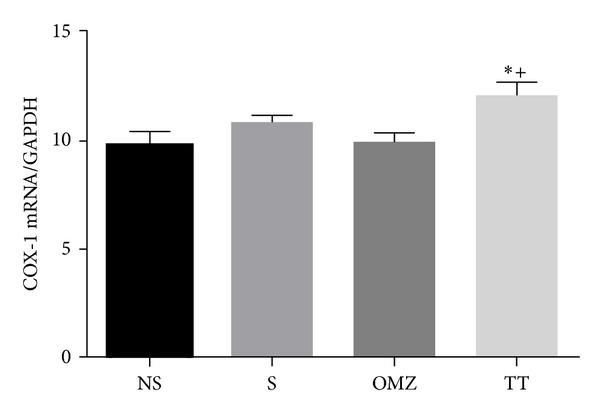
Effects of tocotrienol and omeprazole on gastric COX-1 mRNA expression level in rats exposed to water immersion restraint stress (*n* = 7). *versus nonstressed control (NS) (*P* < 0.05). ^+^versus stressed control (S) (*P* < 0.05).

The ratio of COX-1 mRNA and GAPDH in the intact mucosa was comparable to that of the rats exposed to stress and which developed gastric lesions and rats treated with omeprazole as shown in Figure 5. However, the COX-1 and GAPDH ratio was significantly higher in the tocotrienol-treated group compared to the nonstressed group (*P* = 0.0024) and the stressed control (*P* = 0.0086) (*F*(3,24) = 6.515, *P* = 0.0009), which proposes the ability of tocotrienol to upregulate the COX-1 gene expression in stress. This finding is also well correlated with the increase in PGE_2_ content in the TT group, as shown in Table 1.

**Figure 6:**
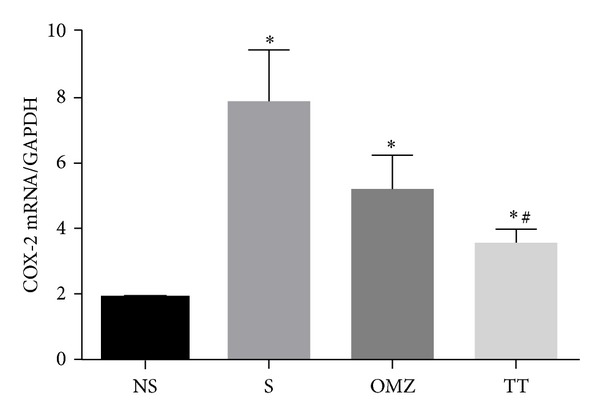
Effects of tocotrienol and omeprazole on gastric COX-2 mRNA expression level in rats exposed to water immersion restraint stress (*n* = 7). *versus nonstressed control (NS) (*P* < 0.05). ^#^ versus stressed control (S) (*P* < 0.05).

The mRNA for COX-2 gene was negligible in the nonstressed rats but increased significantly in all rats exposed to stress (Figure 6). The most significant increase was observed in the nontreated stressed control (*P* = 0.0003), which correlated with the lesions scoring (Table 1) (*F*(3,24) = 7.290, *P* = 0.0004). The COX-2 mRNA gene in the TT group was significantly reduced (*P* = 0.0091) when compared to the stressed control.

## 4. Discussion

Endoscopic studies generally indicate that approximately 75% to 100% of critically ill patients have gross gastric lesions visible when endoscopy is performed within the first 1 to 3 days of illness [21, 22]. The prevalence of stress-related mucosal damage ranges from 15% to 50% if occult bleeding is used as an end point [23]. Morbidity and mortality of patients with critical illness are positively correlated with the degree of oxidative stress [23]. Therefore, the administration of antioxidants such as tocotrienol seems to be a reasonable therapeutic approach. In the present study, gastric lesions developed in the glandular part of gastric mucosa in response to restraint plus water-immersion stress for 3.5 hours. The lesion was in the form of generalized erythema, hemorrhages, and erosion of the mucosa. This result was in agreement with a previous finding where it is important to maintain balance between the destruction and protective capacity of the gastric mucosa [24]. It was previously explained that the increased formation of the gastric lesions might be due to the reduction of blood flow [25], increased acid concentration [26, 27], reduced prostaglandin content [27, 28], and gastric contractions which resulted in temporary restriction of blood flow to the mucosa, producing anoxic damage [28, 29] and free radicals [30, 31]. 

It is well known that gastric mucosa is continuously exposed to harmful factors. In our study, water-immersion restraint stress for 3.5 hours significantly increased the gastric acidity. Similar findings had been reported by Dalia et al. [27] and Konturek et al. [32]. Dalia et al. [27] found that rats exposed to cold-restrain stress for 3.5 hours exhibited a significant increase in acid secretory activity in terms of the acid output, as compared to those of the controls. The finding from the present study showed that the gastric acidity of the tocotrienol (TT) and omeprazole (OMZ) groups was significantly reduced after exposure to WRS compared to the stressed control. Omeprazole is a proton pump inhibitor that suppresses gastric acid secretion by specific inhibition of the H^+^/K^+^-ATPase in the gastric parietal cell. It blocks the final step in acid production, thus reducing gastric acidity. It is of great basis to compare the effect of tocotrienol with omeprazole due to the fact that it is currently one of the commonly prescribed drugs for the treatment of peptic-ulcer disease in a clinical setting. The ability of tocotrienol to reduce gastric acidity in stress could partly be due to its ability to increase PGE_2_ level, which was observed in the current study. 

Prostaglandins, especially PGE_2_, also have cytoprotective effects on gastric mucosa as a consequence of various physiological mechanisms that include increased epithelial mucus and bicarbonate secretions, amelioration of mucosal blood flow and inhibition of free radical activities, and enzyme release from neutrophils [8]. A study by Bregonzio et al. [33] found that stress-induced mucosal ulcerations were also associated with a significant decrease in the gastric mucosal levels of PGE_2_. Our present study showed that gastric PGE_2_ content after 3.5 hours exposure to WRS was significantly suppressed compared to that of the control group. These findings are consistent with previous reports by Konturek et al. [32] and Tanaka et al. [28]. The increased gastric PGE_2_ content in the TT group could possibly be due to the effect of vitamin E, which was reported to stimulate prostaglandin synthesis by activating the calcium-dependent phospholipase enzyme A2 and inhibiting the lipoxygenase enzyme [34]. 

The pharmacotherapy using misoprostol, a synthetic PGE_1_ analog, had shown positive effect against stress-induced gastric injuries when administered at a high dose in patients in ICUs [35]. Although effective, the side effects of diarrhea and abdominal discomfort lead to a low rate of misoprostol use in a clinical setting. The effectiveness of tocotrienol in increasing PGE_2_ could account for its beneficial effect in reducing the risk of gastric ulcers in critically ill patients without the side effect of misoprostol. Although this is true based on the current findings, further studies on humans are warranted to elucidate the effect of tocotrienol on humans and the dosage required for similar effects. 

We also found that the increased COX-1 mRNA expression during stress in the TT-treated group had resulted in the maintenance of the protective PGE_2_ content in the gastric microenvironment. This was not observed in the rats treated with omeprazole. To our knowledge, this finding has never been reported before. It was previously reported that COX-1 generation was observed in gastric mucosa under normal conditions and remained unchanged after exposure to stress [28], as shown in the current study. This proves that COX-1 enzyme is important for gastric protection. Although the mucus and blood flow were not measured in the current study, they were already proved in previous studies, showing that COX-1 enzyme is important in providing the baseline or physiological PGE_2_, which helps maintain the mucus, bicarbonate, and mucosal blood flow [9, 10, 36–38]. Thus, pre-treatment with tocotrienol may prevent gastric mucosal injury caused by stress by increasing the PGE_2_ level.

Interestingly, we also found an increase in COX-2 mRNA expression in rats with gastric lesions formation. Brzozowski et al. [25] suggested that the expression of COX-2 mRNA after water-immersion restraint stress might be due to the deficient of PGE_2_ generation in the gastric mucosa. Thus, this expression might reflect the suppression of PGE_2_ generation because COX-2 plays a crucial role in the healing of gastric ulcers [39]. COX-2, the inducible enzyme, is upregulated by proinflammatory cytokines and growth factors, where it mediates pathological reactions such as inflammation [40, 41]. It was also reported that COX-2 protein is highly localized in fibroblasts, monocytes/macrophages, and granulocytes in the base of gastric ulcers in rats [42]. 

In 2006, Kotani et al. [42] reported that the selective COX-2 inhibitor rofecoxib significantly aggravated the development of gastric lesions in response to ischemia-reperfusion, confirming the involvement of COX-2 in mucosal defence. The findings in this study showed that stressed control rats had the highest rise in COX-2 expression compared to the rats treated with either omeprazole or tocotrienol, and this correlated with the extent of damage to the gastric mucosa. This confirms that COX-2 expression increased in response to injury and that it may play a significant role in gastric repair and the extent of the damage determines the increment of the enzyme expression. Wilankar et al. [43] described the ability of gamma-tocotrienol as an anti-inflammatory agent, where it inhibited the proinflammatory cytokines. The tocotrienol mixture extract from palm oil contained a higher amount of gamma tocotrienol compared to other isomers, which is almost 50%. This could attribute to its ability to reduce proinflammatory cytokines, thus reducing the COX-2 induction as shown in this study. 

Brzozowski et al. [25] found that the exposure to stress led to ischemia reperfusion, which produced a significant fall in PGE_2_ generation in the gastric mucosa, but it was gradually restored during mucosal recovery from gastric lesions, suggesting that endogenous prostaglandin is involved in the spontaneous healing of these lesions. This is supported by the fact that PGE_2_ generation reached higher values during the course of healing of ulcerated gastric mucosa than it did in nonulcerated mucosa. Konturek et al. [6] showed that the healing of stress lesions resulted in the restoration of mucosal prostaglandin generation, and this effect was accompanied by overexpression of EGF and TNF alpha as well as COX-1 and COX-2 mRNA and by the increased biosynthesis of gastroprotective prostaglandins. Takeuchi et al. [44] also found that treatment with 16,16-dimethyl PGE_2_ was able to decrease the mucosal ulcer in rats exposed to stress. Our finding as well as that of others suggests that PGE_2_ seems to be an important determinant in the pathogenesis of stress-induced gastric mucosal lesions. If this is true, then supplementation of tocotrienol may prove to be a good alternative towards reducing stress-induced gastric lesions.

## 5. Conclusion

We conclude that supplementation with tocotrienol protected rats gastric mucosa against stress-induced lesions possibly by reducing gastric acidity and by increasing biosynthesis of gastric PGE_2_ by increasing COX-1 mRNA expression, a desirable factor in the preservation of gastric mucosa integrity. Tocotrienol was found to be better than omeprazole as it was found to prevent gastric mucosal damage caused by stress mainly by reducing gastric acidity. If this is true, supplementation with tocotrienol may prove to be a good alternative towards reducing stress-induced gastric ulcers in critically ill patients. 

## Figures and Tables

**Table 1:** Correlation (*r*) between the parameters studied.

	COX-2/GAPDH mRNA	COX-1/GAPDH mRNA	PGE_2_	Gastric acidity
Lesions index	0.6*	−0.05	−0.4*	0.4*
Gastric acidity	0.35*	−0.3*	−0.4*	
PGE_2_	−0.3*	0.3*		
COX-1/GAPDH mRNA	−0.1			

*Correlation is significant at *P* < 0.05.
